# Phylogenetic Analysis of *Staphylococcus aureus* CC398 Reveals a Sub-Lineage Epidemiologically Associated with Infections in Horses

**DOI:** 10.1371/journal.pone.0088083

**Published:** 2014-02-04

**Authors:** Mohamed M. H. Abdelbary, Anne Wittenberg, Christiane Cuny, Franziska Layer, Kevin Kurt, Lothar H. Wieler, Birgit Walther, Robert Skov, Jesper Larsen, Henrik Hasman, J. Ross Fitzgerald, Tara C. Smith, J. A. Wagenaar, Annalisa Pantosti, Marie Hallin, Marc J. Struelens, Giles Edwards, R. Böse, Ulrich Nübel, Wolfgang Witte

**Affiliations:** 1 Robert Koch Institute, Wernigerode, Germany; 2 Institute of Microbiology and Epizootics, Free University Berlin, Berlin, Germany; 3 Microbiology and Infection Control, Statens Serum Institut, Copenhagen, Denmark; 4 National Food Institute, Technical University of Denmark, Lyngby, Denmark; 5 The Roslin Institute, University of Edinburgh, Edinburgh, United Kingdom; 6 Department of Epidemiology, College of Public Health, the University of Iowa, Iowa City, Iowa, United States of America; 7 Department of Infectious Diseases and Immunology, Faculty of Veterinary Medicine, Utrecht University, Utrecht, the Netherlands; 8 Istituto Superiore di Sanità, Rome, Italy; 9 Centre National de Référence *Staphylococcus aureus*, Microbiology Department, Erasme University Hospital, Université Libre de Bruxelles, Brussels, Belgium; 10 European Centre for Disease Prevention and Control, Stockholm, Sweden; 11 Department of Microbiology, Scottish MRSA Reference Laboratory (SMRSARL), Glasgow Royal Infirmary, Glasgow, United Kingdom; 12 Labor Dr. Böse GmbH, Harsum, Germany; National Institutes of Health, United States of America

## Abstract

In the early 2000s, a particular MRSA clonal complex (CC398) was found mainly in pigs and pig farmers in Europe. Since then, CC398 has been detected among a wide variety of animal species worldwide. We investigated the population structure of CC398 through mutation discovery at 97 genetic housekeeping loci, which are distributed along the CC398 chromosome within 195 CC398 isolates, collected from various countries and host species, including humans. Most of the isolates in this collection were received from collaborating microbiologists, who had preserved them over years. We discovered 96 bi-allelic polymorphisms, and phylogenetic analyses revealed that an epidemic sub-clone within CC398 (dubbed ‘clade (C)’) has spread within and between equine hospitals, where it causes nosocomial infections in horses and colonises the personnel. While clade (C) was strongly associated with *S. aureus* from horses in veterinary-care settings (p = 2×10^−7^), it remained extremely rare among *S. aureus* isolates from human infections.

## Introduction

*Staphylococcus aureus* is a frequent nasal coloniser of mammals and birds. In humans, it is a leading cause of a wide range of infections in hospitals and communities. In particular, infections caused by methicillin-resistant *S. aureus* (MRSA) are of special concern due to the limited treatment options [1]. In addition to being a major threat to human health, since the 2000s MRSA is widely disseminated as a coloniser and infectious agent in economically important livestock and companion animals including cows, sheep, goats, poultry, pigs, dogs and horses. The first sporadic reports of MRSA infections in livestock arose during the 1970s and in companion animals (dogs and cats) during the late 1980s and 1990s [2]–[4].

In the early 2000s, a new clonal complex of MRSA (CC398) was detected in pigs in the Netherlands [5]. Since then, CC398 has been the dominant livestock-associated MRSA (LA-MRSA) among pigs in several countries [6]-[11], but CC398 has also been found in various other livestock species [12]–[20]. The transmission of CC398 from pigs to pig farmers has been reported previously [5], [6], [21]–[24]. Hence, direct contact with livestock is considered a risk factor for human colonisation and infection with CC398 [25]. However, several studies have reported human cases of methicillin-sensitive CC398 without current contact with livestock [21], [26], [27].

A previous study suggested that CC398 originated in humans as MSSA and was subsequently transmitted to livestock, where it then acquired the methicillin resistance [28]. In addition to livestock, CC398 has been recovered from companion animals and other animal species [6], [22], [29], [30]. For instance, CC398 has been isolated from horses in Austria, Belgium, Germany, the United Kingdom and the Netherlands [6], [31]–[36]. Nosocomial spreading and infection with MRSA in veterinary hospitals have been described previously [31], [37]. Several infection cases, outbreaks, and colonisations of horses and associated personnel with CC398 have been reported in equine hospitals from several countries [23], [31], [35], [38]–[40].

In this study, we used mutation discovery to elucidate the population structure and evolution of MRSA CC398 from infections in horses in comparison to a collection of isolates from other host species originating from various countries in Europe and overseas. We demonstrate that a specific sub-lineage of CC398 has emerged in equine veterinary care.

## Results and Discussion

### Molecular typing

In this study, a convenience sample collection of 195 *S. aureus* isolates, including MSSA (n = 37) and MRSA (n = 158), was investigated (Table 1). Isolates were collected between 1993 and 2011 from twelve different host species in ten different countries (Table S1). Molecular typing of the 195 isolates revealed fourteen different *spa* types (t011, t034, t108, t571, t779, t899, t1197, t1344, t1451, t2576, t2876, t2974, t5972 and t6867) (Table S1). *Spa* types t011 and t034 were the most common, representing 45% and 40% of the isolates, respectively. Furthermore, approximately 50% of the isolates (n = 99) harboured SCC*mec* type V, while 27% of the isolates (n = 52) carried SCC*mec* type IV (Table S1).

**Table 1:** Summary of the isolate collection investigated in this study.

Country of origin	Host species	Year of isolation	Colonisation/Infection
Austria (n = 17)	Bovine (n = 6)	1993 (n = 1)	Colonisation (n = 29)
Belgium (n = 6)	Cat (n = 1)	2001 (n = 1)	Infection (n = 72)
Canada (n = 1)	Chicken (n = 7)	2002 (n = 2)	Information not available (n = 94)
Denmark (n = 31)	Dog (n = 5)	2003 (n = 3)	
Germany (n = 110)	Environment (n = 1)	2004 (n = 7)	
Italy (n = 3)	Goat (n = 1)	2005 (n = 5)	
The Netherland (n = 15)	Goose (n = 2)	2006 (n = 11)	
Thailand (n = 1)	Horse (n = 53)	2007 (n = 53)	
UK (n = 5)	Human (n = 80)	2008 (n = 31)	
USA (n = 6)	Pig (n = 35)	2009 (n = 33)	
	Turkey (n = 4)	2010 (n = 12)	
		2011 (n = 38)	

### Phylogeny

We used denaturing high-pressure liquid chromatography (dHPLC) for mutation discovery at 97 genetic housekeeping loci (≈400 bp per locus) distributed along the *S. aureus* chromosome; in total, they constituted 1.4% (40,230 bp) of the CC398 genome.

Our analysis revealed 96 bi-allelic polymorphisms (i. e., polymorphic sites at which two alleles were observed) associated with 63 haplotypes. Among these polymorphisms were 34 synonymous point mutations in the protein coding genes, 58 non-synonymous point mutations, and 4 insertions or deletions ranging in size from 1 to 14 bp (Table S2). Of these, 41 polymorphisms were informative for maximum parsimony analyses. The nucleotide diversity, π (the average number of nucleotide dissimilarities per site among two isolate sequences), was 0.00008±0.00001 for the coding regions. The mean nucleotide substitution rate was estimated at 5.4×10^−6^ substitutions/nucleotide site/year (95% confidence interval, 3.5×10^−6^ to 7.5×10^−6^). This estimated mutation rate for the isolate collection is relatively faster than a previously reported evolutionary rate for other *S. aureus* strains [41], [42]. To investigate the time of the most recent common ancestor (TMRCA) of the 195 CC398 isolates, we applied a Bayesian coalescent method of phylogenetic inference as previously described [41]. According to the calculated mutation rate, the sequences variations and the isolation date (1993–2011) of our isolates dataset, we estimated that the TMRCA was ≈1974 (95% confidence interval, 1955 to 1991).

**Figure 1:**
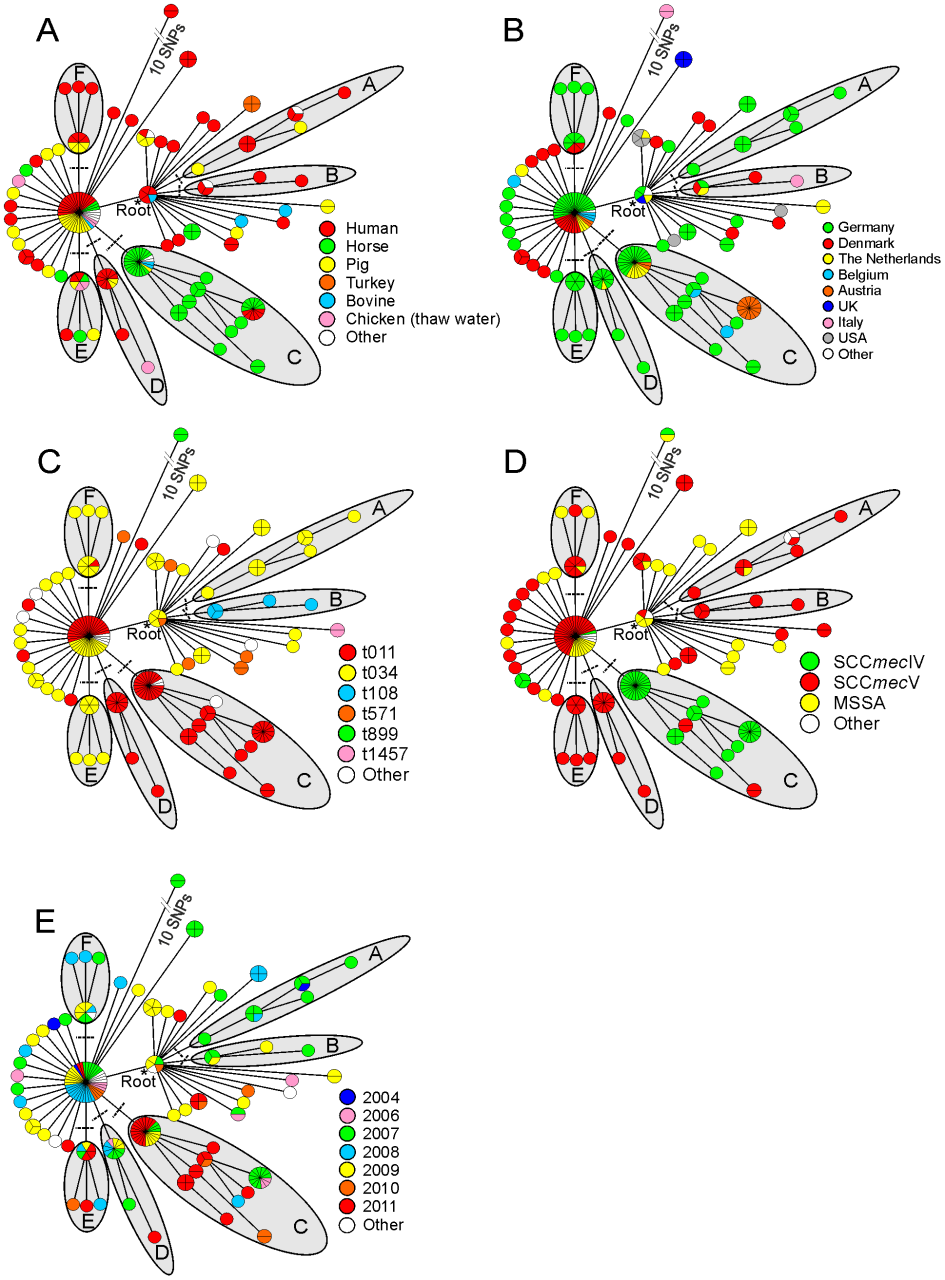
Minimum spanning tree (MST) represent the clustering of 195 CC398 isolates based on genome-wide SNPs; The ancestral node was determined by comparing concatenated sequences from the investigated loci of all investigated CC398 isolates with the concatenated sequences of N315 as an out-group. A) host origin of the 195 CC398 isolates, B) plotting of the geographical distribution on the MST, C) shows the different *spa* types, D) the SCC*mec* types and the susceptibility of the 195 CC398 isolates and E) the isolation date.

Based on these 96 polymorphisms, a minimum spanning tree (MST) was constructed (Figure 1). The MST demonstrated very limited diversity among the 195 investigated isolates. The ancestral node was determined by comparing concatenated sequences from the investigated loci of all investigated CC398 isolates with the concatenated sequences of N315 as an out-group. Rooting the phylogenetic tree of CC398 using N315 as an out-group revealed that isolates with *spa* type t899 (n = 2) were the most divergent group in comparison with the remaining CC398 isolates (Figure 1C). The t899 isolates had 10 mutations compared to the root (au200-2, au200-3, au201-1, au201-2, au201-3, au202-1, au202-2, au202-3, au202-4 and au202-5), which were located on the isolates chromosomes within a region of ≥111,139 bp (between 23,209 -134,348) (Table S2). This finding is in agreement with a study based on whole genome sequencing, which suggested that CC398 with *spa* type t899 had acquired a fragment of 123,000 bp from ST9 through horizontal gene transfer. This fragment included the *spa* gene and the SCC*mec* insertion site [28].

### Correlation of certain *spa* types and SCC*mec* types with phylogenetic lineages of CC398

The MST revealed six main clades (A to F) within CC398 (Figure 1) (mutations defining each clade are listed in table S2). Mapping the 14 *spa* types and the SCC*mec* types onto the MST revealed that clade (B) consisted of isolates (n = 5) from different countries that shared the same *spa* type (t108), and four of them carried SCC*mec* type V (Figure 1C & D). Similarly, clades (A, E, and F) were composed entirely of isolates characterised by *spa* type t034, with the exception of one isolate within clade (F), which was represented by *spa* type t011 (Figure 1C). Furthermore, the Bayesian tip-association significance test (BaTS) [43] revealed that certain *spa* types (t034, t011, t571, t108, t1457, and t899), and SCC*mec* types (IV, and V) were significantly associated with phylogeny (p<0.01; Table S3). Nevertheless, our findings confirm that the usage of *spa* sequencing as the single typing tool for *S. aureus* might occasionally lead to misinterpretation, which is in agreement with previous observations in similar studies of other clonal complexes of *S. aureus* [28], [41], [44], [45].

### Association of host origin with phylogeny

Our analysis included CC398 isolates from 11 host species. A Bayesian statistical test (BaTS analysis; [43]) indicated that among these isolates, those from turkey meat and those from horses each displayed a significant association with phylogenetic structure within CC398 (p<0.01; Table S3). In contrast, other host species including humans were more dispersed on the phylogenetic tree (Figure 1A), not significantly different from a random distribution (p>0.1; Table S3).

The number of turkey isolates in our collection was very small (n = 4), and their geographic origins are not known with precision. Interestingly, however, we detected the φAvβ prophage in these four isolates by using targeted PCR (Table S1). This prophage was previously reported from *S. aureus* from several other bird species, suggesting CC398 in turkey may have adapted to the host through acquisition of an avian-specific prophage, similar to *S. aureus* CC5 in industrially fattened broiler chickens [46].

It is remarkable that the majority of isolates from horses under veterinary care clustered in clade (C) (41 out of 53 equine isolates total; Figure 1A). Of note, human isolates in the same clade (n = 6; 07-00334, 07-00471, 07-01238, 07-01239, 07-01335, 07-01730) were from veterinary personnel of an equine clinic in a large Austrian veterinary hospital (Stationary Care 1) who had close contact to infected horses (Table S1) [31]. Isolates in clade (C) (n = 53) had been collected from four different countries (Austria, Belgium, Germany, and the Netherlands) between 2006 and 2011 (Figure 1E), and clade (C) isolates from Germany (n = 29) had been collected from 13 equine clinics and veterinary practitioners distributed over seven different federal states (Table S1). These findings imply that clade (C) within CC398 is disseminated among hospitalised horses and veterinary personnel all over Germany and in several neighbouring European countries. At the same time, we found that clade (C) was extremely rare among *S. aureus* isolates from human infections in Germany. Among >6,700 isolates that had been submitted to the German National Reference Centre for Staphylococci and Enterococci in Robert Koch Institute between 2010 and 2011, there were 48 MRSA from human infections that displayed *spa* type t011 (Table S4). Among these, only four isolates carried the synonymous base substitution that defines clade (C) (i. e., they carried a thymidine residue at genomic position 2,533,404; SNP au309-2; Tables S2, S4), as revealed by targeted PCR and sequencing. Hence, the association of clade (C) with infections in horses is highly significant (p<0.0001; chi^2^ test).

We assume that the emergence of MRSA CC398 clade (C) in horses from different European equine clinics may be due to epidemic spread, possibly comparable to several epidemic MRSA strains that rapidly spread within and between medical care hospitals and cause a large number of health-care-associated infections in humans [47]–[49]. A previous study based on multilocus sequence typing (MLST), *spa*-typing, and SCC*mec*-typing demonstrated that MRSA-CC398-t011-IV caused nosocomial infections in horses in an equine clinic in Switzerland [36]. The authors reported that MRSA-CC398-t011-IV was first detected in one of the personnel members who formerly worked in an equine clinic in Belgium. Later, this CC398-t011-IV was detected in infected horses and subsequently replaced ST1-t2863, which was prevalent in wound infections in this equine clinic [36]. While samples from Switzerland were not available to us, it is well possible that the strain in this clinic was affiliated to clade (C), since the majority of clade (C) isolates in our collection also displayed *spa* type t011 (95%) and SCCmecIV (91%), respectively (Table S1, Figure S1).

One possible explanation for the spread of this CC398 sub-clone may be insufficient hygiene practices in veterinary settings; however, this requires further research. Several studies have reported that the nasal carriage rate of MRSA among veterinary practitioners is much greater than in medical staff in human hospitals [50]–[54]. In addition, the nosocomial spread of MRSA in equine clinics and between veterinary personnel was previously demonstrated [55]–[59]. Hence, personnel in veterinary settings may play an important role in the introduction and spread of MRSA into equine clinics. In addition, humans with frequent contact with horses can represent a reservoir for MRSA and subsequently transmit it to their household. A metapopulation model demonstrated that the occurrence of a relatively large proportion of MRSA-CC398 carriers among a susceptible human population might result in an outbreak [60].

Of note, the association of equine origin with a phylogenetic clade within CC398 observed here does not immediately indicate any specific genetic adaptation. Such adaptation is difficult to detect in general. Even for healthcare-associated MRSA, which have been studied for decades and for which abundant genome sequence data is available, it has proven extremely difficult to identify the specific adaptive traits that render these strains successful [47], [61], [62]. Notably, clade (C) contained isolates from other hosts (e.g. human (Stationary Care 1); calf, dog, and pig (Farm 1)) who had been in contact with horses, suggesting that genetic specialisation to the equine host may be limited or lacking.

### Limitations of this study

Although our collection of *S. aureus* CC398 isolates represents the broadest host species coverage studied to date, its composition is fragmentary with respect to both, the spatial and temporal coverage of the global population of CC398. While we have taken considerable efforts to assemble a broadly representative strain collection, it includes convenience isolates that a limited number of collaborating microbiologists had considered worth to be preserved over years for various reasons. For example, even though our equine isolates had been collected in several European countries between 2006 and 2011, they by no means represent the demographics of the underlying horse population, let alone that of the more widely distributed population of *S. aureus* CC398. Several other categories (country of origin, host species) contained very few samples (e. g. only one isolate from Canada, one from a cat, etc.). In addition, very limited clinical and other meta-data was available for many of the isolates, because they initially had not been stored with the goal of any global epidemiological inferences in mind. Hence, for an in-depth investigation of the distribution of CC398 among different host species, it would be highly desirable to extend this study by including additional isolates from each of the various hosts, with an even distribution over several years and over a large geographic area, and by systematically recording epidemiological data.

The dHPLC-based mutation discovery method applied here covered 1.4% of the CC398 genome. This approach delivered improved discriminatory power compared to *spa* typing and standard MLST [44], [63], and provided some novel insights into *S. aureus* population structure. However, the resolution of analyses and the strength of any inferences would be much improved by whole-genome sequencing [42], [47].

#### Conclusions

Our study demonstrated new insight into the phylogeny of CC398 through mutation discovery. We revealed the spread of a specific MRSA-CC398 sub-clone ‘dubbed clade (C)’ within equine settings, which causes infections in horses and nasal colonisation of humans. Furthermore, the spread of this sub-clone (clade (C)) can be traced through testing for the presence/absence of SNP (309-2) using diagnostic PCR followed by sequence analysis [64] [65] Veterinarians play an important role in controlling the transmission of this sub-clone by taking precautions with staff hygiene, and implementation of control protocols for infections.

## Materials and Methods

### Bacterial isolates

In the present study, a collection of 195 *S. aureus* CC398 (MSSA; n = 37 and MRSA; n = 158) isolates was investigated (Table S1) and some of these isolates were included in previous studies [26], [28], [31], [51], [66]–[69]. CC398 convenience isolates were collected from nine different countries (mainly from Europe), and various hosts (humans: n = 80; animals: n = 115). The isolates investigated in this study were selected by animal species, geographical origin, and approximate period of time. Veterinary care facilities in this study were divided into stationary care (where the animals must be hospitalized for at least one night in order to receive medical treatment) or ambulatory care (medical care is provided to animals without being admitted to a hospital for treatment). MRSA isolates were chosen as follows:

[1] 10 isolates from horses were collected in Austria/Vienna. Eight of these isolates were from infected horses treated in Vienna veterinary hospital (Stationary Care 1; Table S1) from 2006 until 2007. We had previously collected and investigated isolates from nasal colonization of the veterinary personnel of this hospital, due to the emergence of CC398 over a long period in this facility, [31]. These human isolates (n = 6) were also included.

[2] 37 clinical isolates from horse were collected in Germany, from 17 different veterinary facilities (3 stationary care, 14 ambulatory care), which were distributed over 6 different German federal states (Baden-Württemberg (1), Hesse (1), Lower Saxony (3), North-Rhine-Westphalia (9), Schleswig-Holstein (2), Saxony (2), and Saarland (1)). Most of these horse isolates were sent for typing to the German Reference Centre for Staphylococci and Enterococci in Robert Koch Institute - branch Wernigerode by the Labor Dr. Boese which is providing diagnostic service for veterinarians treating horses in all the German federal states. Isolates from other animal species from Germany were also included, which originated from nasal colonization in pigs, pig farmers and their family members; colonization of posterior nares of goose, broiler chicken carcasses (thawing liquid). Isolates causing mastitis in cattle as well as various kinds of infections in humans emerging at different geographical locations in Germany were included as well (Table S1).

[3] Finally, isolates from other European countries (e.g. Belgium, Denmark, Italy, the Netherlands, UK) as well as from overseas (Canada, Thailand, USA) were included to maximize geographic distribution and range of host species. Additionally, to monitor the dissemination of one particular *S. aureus* strain among different animal species, six isolates from a Dutch farm derived from horses, dog, and cattle were included (farm 1 in Table S1).

SCC*mec*- and *spa*-typing were performed for all isolates as previously described [70]. Briefly, *spa*-typing was performed by following the Ridom Staph Type standard protocol and the *spa*-types were assigned to the Ridom database (www.ridom.org) (Ridom GmbH, Würzburg, Germany). In addition, antimicrobial susceptibility was tested using the broth dilution method according to the DIN58940 guidelines.

### Mutation discovery using dHPLC

In this study, we investigated mainly metabolic housekeeping genes because polymorphisms in these genes provide the most reliable phylogenetic markers [71]. In total, we investigated 97 genetic housekeeping loci, which made up 1.4% (40,230 bp) of the CC398 genome and were scattered over the core genome of CC398. These loci had been analysed previously to investigate the population structure of other clonal complexes of *S. aureus* [41], [44], [72]. PCR primers were used to amplify 97 genetic housekeeping loci distributed along the 195 *S. aureus* isolate chromosomes (Table S2). Mutation discovery for the amplified gene fragments was performed using dHPLC (WaveR Nucleic Acid Fragment Analysis System, Transgenomic, Inc., Omaha, NE, USA) as described previously [44], [46], [72], [73]. Briefly, PCR amplicon from each isolate was compared to a reference strain for detecting the heteroduplexes. Heteroduplexes result in double-stranded DNA that contains a point mutation site in comparison to the reference strain. Identified SNPs were confirmed through capillary Sanger sequencing of the PCR products from both ends using the PCR primers which are listed in Table S2.

### Bacteriophage identification

For identification of phages possessing integrase group φSa3, we performed PCR using the primers int3, f2: 5′GTCAGCTTTAGATGACGC and int3, r2: 5′AGCGCTAATGATGAACGA according to NC_00227452. For PCR demonstration of *sak*, *chp* and *scn*, we followed the protocol as described previously [74]. The presence of φAvβ prophage was determined by PCR as previously described [46].

### Data analysis

Based on the discovered SNPs within the 97 genetic loci, a minimum spanning tree was constructed using Bionumerics software version 6.5 (Applied Maths, Ghent, Belgium). Additionally, sequences from the 97 housekeeping genes were concatenated for each isolate, constituting a 40,230 bp sequence alignment. A maximum likelihood tree based on this alignment was assembled using PhyML 3.1 [75]. The ancestral node was distinct by including distantly associated *S. aureus* genomic sequences. DnaSP was used to estimate the nucleotide diversity (π) and nucleotide variation (θw) and for calculating the mean pair-wise distance between alleles at synonymous (Ks) and non-synonymous (Ka) sites [76]–[78]. The rate of evolution and the divergence times were estimated as described previously using BEAST software (Version 1.7.5, http://beast.bio.ed.ac.uk/) [79]. The Bayesian tip-association significance test (BaTS, version 1.0) was applied to estimates of the association of the phylogeny traits with hosts, *spa* types, geographical origin, and SCC*mec* types [43].

Statistical significance of the association between SNP 309-2 and the host species was assessed using a chi-square test (http://www.r-project.org/).

## Supporting Information

Figure S1 **Maximum-likelihood phylogenetic tree of the 195 CC398 isolates, rooted by comparing concatenated sequences from the investigated loci of all investigated CC398 isolates with the concatenated sequences of N315 as an out-group.**
(PDF)

Table S1 **Bacterial isolates.**
(XLSX)

Table S2 **Genetic loci with their PCR primers and their polymorphisms.**
(XLSX)

Table S3 **Bayesian tip-association significance testing (BaTs) analysis.**
(XLSX)

Table S4 **Additional human isolates checked for the SNP au309_2.**
(XLSX)
