# Supplementary material for: Phylogenetic Analysis of Staphylococcus aureus CC398 Reveals a Sub-Lineage Epidemiologically Associated with Infections in Horses
Source: PLoS One. 2014 Feb 4;9(2):e88083. doi: 10.1371/journal.pone.0088083 (PMC3913741; doi:10.1371/journal.pone.0088083)
Supplement: Figure S1 — Maximum-likelihood phylogenetic tree of the 195 CC398 isolates, rooted by comparing concatenated sequences from the investigated loci of all investigated CC398 isolates with the concatenated sequences of N315 as an out-group. (PDF) [file pone.0088083.s001.pdf]

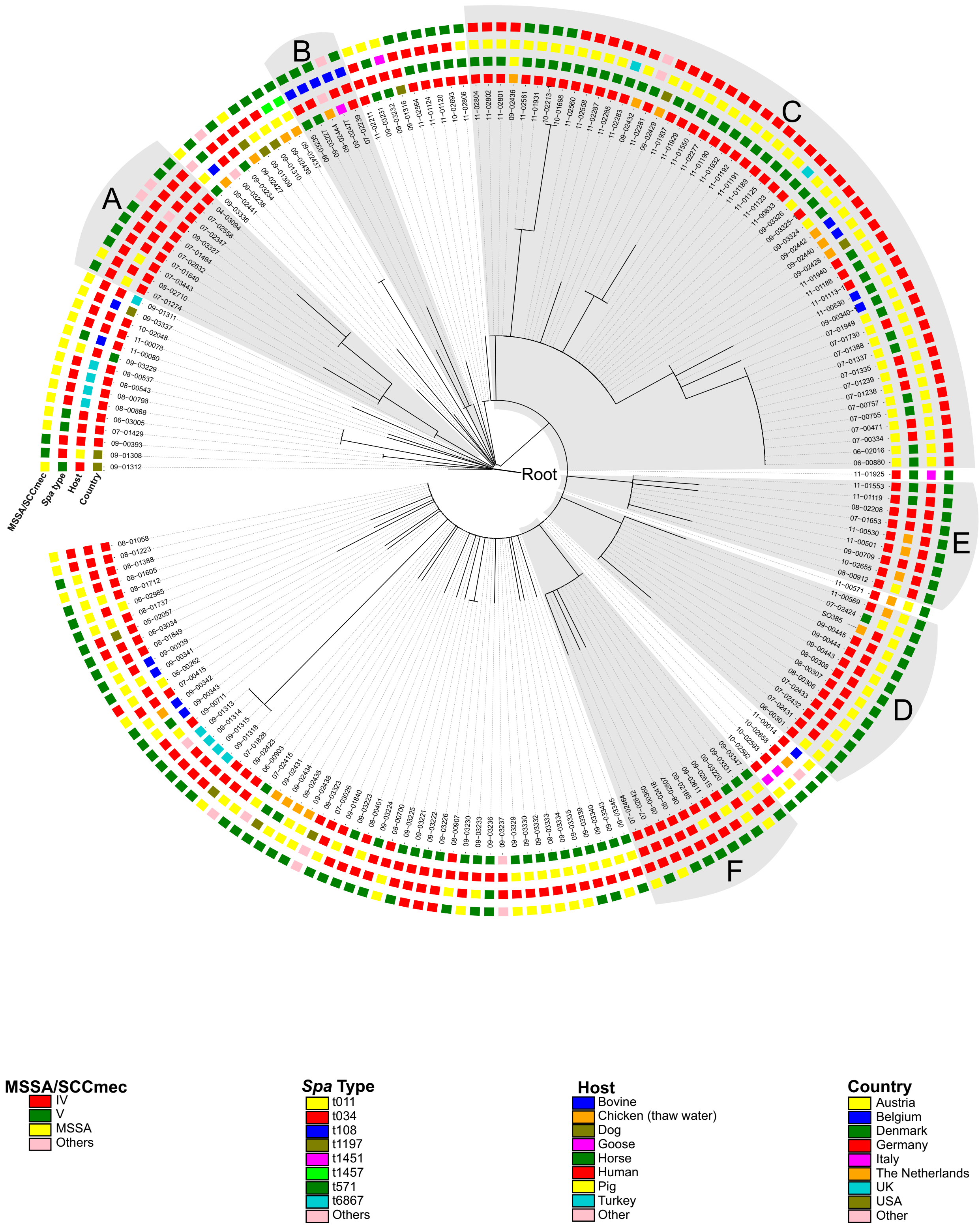
A
B
C
D
E
F
Root
MSSA/SCCmec
Spa type
Host
Country
MSSA/SCCmec
IV
V
MSSA
Others
Spa Type
t011
t034
t108
t1197
t1451
t1457
t571
t6867
Others
Host
Bovine
Chicken (thaw water)
Dog
Goose
Horse
Human
Pig
Turkey
Other
Country
Austria
Belgium
Denmark
Germany
Italy
The Netherlands
UK
USA
Other
